# 3D TiO_2_@Ni(OH)_2_ Core-shell Arrays with Tunable Nanostructure for Hybrid Supercapacitor Application

**DOI:** 10.1038/srep13940

**Published:** 2015-09-10

**Authors:** Qingqing Ke, Minrui Zheng, Huajun Liu, Cao Guan, Lu Mao, John Wang

**Affiliations:** 1 Department of Materials Science and Engineering, National University of Singapore, Singapore 117574; 2 Department of Physics, National University of Singapore, 2 Science Drive 3, Singapore 117542; 3 Institute of Materials Research and Engineering, A*STAR (Agency for Science, Technology and Research), Singapore 11760

## Abstract

Three dimensional hierarchical nanostructures have attracted great attention for electrochemical energy storage applications. In this work, self-supported TiO_2_@Ni(OH)_2_ core-shell nanowire arrays are prepared on carbon fiber paper via the combination of hydrothermal synthesis and chemical bath deposition. In this core-shell hybrid, the morphology and wall size of the interconnected nanoflake shell of Ni(OH)_2_ can be tuned through adjusting the concentration of ammonia solution. Heterogeneous nucleation and subsequent oriented crystal growth are identified to be the synthesis mechanism affecting the nanostructure of the shell material, which consequently determines the electrochemical performance in both energy storage and charge transfer. Superior capabilities of 264 mAhg^−1^ at 1 A g^−1^ and 178 mAh g^−1^ at 10 A g^−1^ are achieved with the core-shell hybrids of the optimized structure. The asymmetric supercapacitor prototype, comprising of TiO_2_@Ni(OH)_2_ as the anode and mesoporous carbons (MCs) as the cathode, is shown to exhibit superior electrochemical performance with high energy and power densities. The present work provides a clear illustration of the structure-property relationship in nanocrystal synthesis and offers a potential strategy to enhance the battery type Ni(OH)_2_ electrode in a hybrid supercapacitor device.

Three dimensional (3D) core-shell nanoarrays grown on substrates have attracted great scientific and technological attentions due to their high versatility and applicability as the essential components in nanoscale electronics, catalysis, chemical sensors, and energy conversion storage devices^1^,^2^,^3^,^4^. In comparison to bulk materials, core-shell heterostructures not only provide a large interfacial area between electrode and electrolyte for charge transport and shortened diffusion path for intercalation/de-intercalation of active species, but also give rise to synergetic properties or multifunctionalities provided by individual components. Therefore, a novel constructions of core-shell structures are needed for fabrication of high-performance electrochemical energy storage devices. Currently, considerable efforts have been devoted to explore various core-shell structures, including metal/metal oxide, metal/metal, metal oxide/metal oxide and metal oxide/conductive polymer^5^,^6^. Transition metal oxides and hydroxides (e.g., Co_3_O_4_, Co(OH)_2_, MnO_2_, Mn(OH)_2_, NiO, Ni(OH)_2_) acting as branched shell materials were generally integrated with conducting scaffold into core-shell structures (e.g., CoO@Co(OH)_2_, CNT@Ni(OH)_2_, SnO_2_@MnO_2_)^7^,^8^,^9^,^10^.

TiO_2_ is an inexpensive and electrochemically stable semiconductor commonly used in lithium batteries^11^. Moreover, the electrical conductivity of around 10^−5^–10^−2^ S cm^−1^ permits its application as backbone in the core-shell structure (e.g., TiO_2_@MnO_2_, TiO_2_/NiO)^12^,^13^. To enhance the storage capability of TiO_2_-based hybrids, strategies have been focused on enhancing the conductivity of TiO_2_^14^. For example, Lu *et al.* managed to increase the electrical conductivity of TiO_2_ by three orders of magnitudes through hydrogenation^11^. Alternatively, Liao *et al.* improved the electrical conductivity through coating carbon across the outer layer of TiO_2_^12^. These efforts to some extent have boosted the storage capacity, rate capability and cyclability in energy storage. However, few studies have been made in optimizing the shell structure, especially in controlling the shell configuration and engineering the interface, which are the key parameters in affecting the electrochemical performance.

There are an increasing number of studies regarding active electrode materials that undergo faradaic reactions but are used for electrochemical capacitor applications. Unfortunately, some of these materials are described as “pseudocapacitive” materials despite the fact that their electrochemical signature (e.g., cyclic voltammogram and charge/discharge curve) is analogous to that of a “battery” material, as commonly observed for Ni(OH)_2_ in KOH electrolyte^15^,^16^. Ni(OH)_2_ has been the focus of research interest, owing to its high stability, low toxicity, low cost and high theoretical capacity, which make it useful for energy storage^17^,^18^,^19^,^20^,^21^. For freestanding Ni(OH)_2_ particles, the electrochemical performance was reported to highly correlate with several nanostructure parameters, including crystallite size, structural defects and surface area^21^,^22^. Ni(OH)_2_ particles in the form of hollow microsphere-like, nanoflower-like, and nanoplate-like configuration show strong structure-property relationship^23^,^24^,^25^. Therefore, successful synthesis of Ni(OH)_2_ with desired nanostructures is of paramount importance. In the present work, we have designed a new 3D structure of TiO_2_@Ni(OH)_2_ core-shell nanoarrays grown on carbon fiber paper (CFP). Interestingly, the nanostructure of Ni(OH)_2_ branches can be greatly modified by adjusting the concentration of ammonia added in the solution mixture. Superior electrochemical performances of 264 mAh g^−1^ at 1 A g^−1^ and 178 mAh g^−1^ at 10 A g^−1^ are achieved with the core-shell hybrid of optimized structure. The structure-property relationships observed for the core-shell nanoarrays in this work offer a new strategy to enhance the performance of supercapacitor electrodes.

## Results

**Figure 1:**
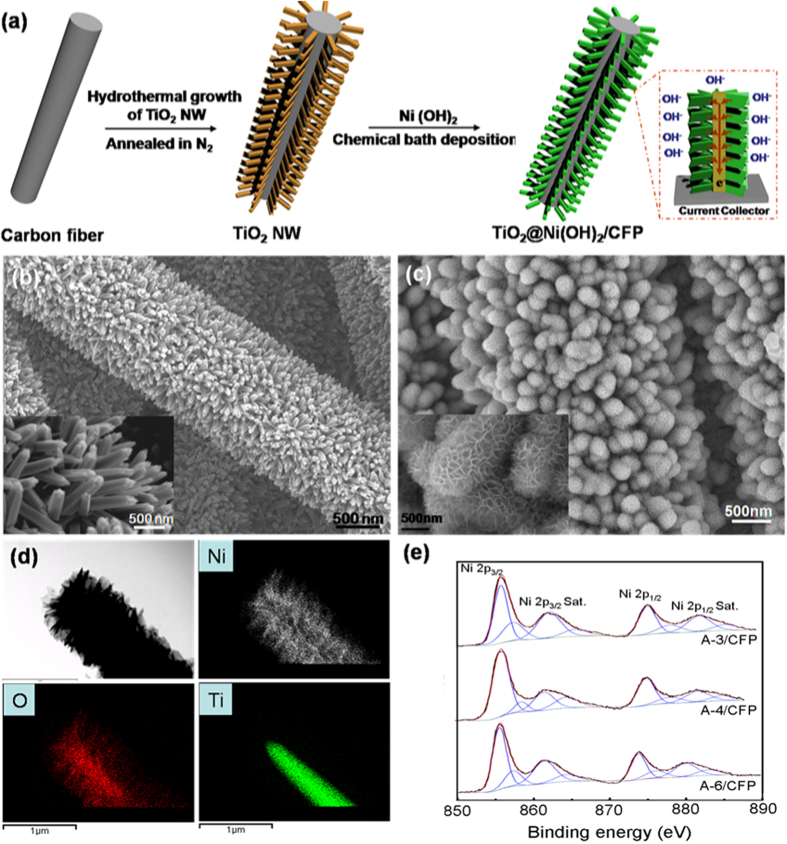
(**a**) Schematic illustration for the fabrication of TiO_2_@Ni(OH)_2_ core-shell nanowire arrays on CFP. (**b**) SEM image of the TiO_2_ nanowire arrays, and (**c**) TiO_2_@Ni(OH)_2_ core/shell nanowire arrays on the CFP. The fine structures are shown in insets. (**d**) EDS mapping results from a single hybrid nanowire, demonstrating the TiO_2_ core and Ni(OH)_2_ shell hierarchical structure. (**e**) XPS spectroscopy of Ni 2p in samples of A-3/CFP, A-4/CFP and A-6/CFP.

The fabrication procedures of the core-shell nanowire electrodes are illustrated in Figure 1a. In the typical preparation route, the TiO_2_ nanowires were prepared on a CFP in the form of branched nanowire arrays by hydrothermal method. In the following step, metal hydroxide nanoflakes of Ni(OH)_2_ are grown onto the TiO_2_ nanowires by a chemical bath deposition. During this process, different amounts of aqueous ammonia (24% NH_3_·H_2_O, 3ml, 4ml, 6ml) are added to the solution, and the corresponding samples are labeled as A-3/CFP, A-4/CFP, A-6/CFP, respectively (see the Experimental Section). In the 3D core-shell hierarchical nanostructure, the core material is directly aligned on the current collector. This rationalized configuration is to provide both large active surface areas and good electrical connection for fast redox kinetics, therefore being capable of boosting the efficiency of the energy storage^7^. In the present work, the surface of carbon fibers was largely covered by the TiO_2_ nanowires, which have diameters of ~200 nm and grown nearly vertical to the carbon fiber forming nanowires arrays (Fig. 1b). After the chemical bath deposition process, the interconnected Ni(OH)_2_ nanoflakes fully decorate the scaffold of TiO_2_ nanowires, giving rise to a core-shell configuration with diameters of ~1000 nm (Fig. 1c). A single hybrid nanowire of TiO_2_@Ni(OH)_2_ was unambiguously examined by the energy dispersive X-ray spectrometry (EDS) mapping analysis (see Fig. 1d), which indicates that the inner nanowire core is TiO_2_ and the outer-layer shell is Ni(OH)_2_ in the hybrid core-shell structure. To demonstrate the effect of the amount of ammonia on the likely change of chemical states in Ni ion, XPS survey of Ni ion is collected as shown in Fig. 1e. All the Ni XPS surveyed in the three samples show two major peaks centered at 856.1 and 873.6 eV with a spin-energy separation of 17.5 eV, corresponding to Ni 2p_3/2_ and Ni 2p_1/2_, respectively, together with extra three sets of peaks located at higher energy sides being correlated with the Ni 2p_3/2_ and Ni 2p_1/2_ satellite peaks^26^. These bands are characteristic peaks of Ni(OH)_2_ phase, which is consistent with previous reports^27^.

**Figure 2:**
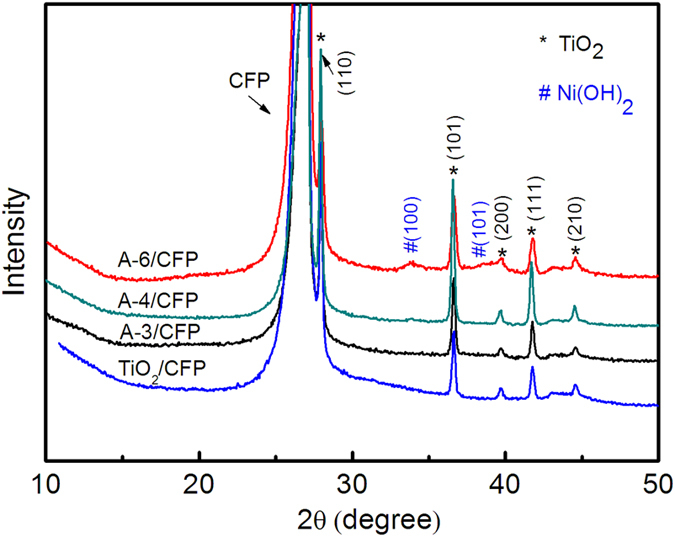
XRD patterns of the A-3/CFP, A-4/CFP, A-6/CFP and TiO_2_/CFP nanowire electrodes.

The XRD patterns of the A-3/CFP, A-4/CFP, and A-6/CFP nanowire electrodes are shown in Fig. 2, where the diffraction pattern of the TiO_2_/CFP is also collected as a reference for comparison. The diffraction peaks positioned at 2θ of 27.9^o^, 36.6^o^, 40.01^o^, 41.7°, 44.4^o^ assigning to (110), (101), (200), (111) and (210) respectively, confirm the rutile phase of the TiO_2_ nanowires. In addition to the diffraction peaks from TiO_2_, peaks of low intensity arising from Ni(OH)_2_ are also collected, as a result of the poor degree of crystallization. Moreover, one notes that compared with the diffraction pattern in A-3/CFP and A-4/CFP, two small humps located at 33.7^o^ and 38.4^o^ were observed for A-6/CFP, which were assigned to (100) and (101) of β-Ni(OH)_2_, respectively (JCPDS card no. 1-1047)^28^. Therefore, the addition of more ammonia tends to increase the crystallinity of Ni(OH)_2_ in the chemical bath deposition process, although concentrations of other salts in the solution are also changed.

Table 1 summarizes the BET surface area and BJH desorption cumulative pore volume results for TiO_2_/CFP, A-3/CFP, A-4/CFP and A-6/CFP electrodes. The BJH desorption cumulative pore volume is calculated on the basis of pores sizes from 1.7 to 300 nm^28^. For the TiO_2_/CFP, the BET surface area is 2.56 m^2^ g^−1^. After chemical deposition of Ni(OH)_2_ on the TiO_2_/CFP, the surface area and pore volume are tremendously increased from 2.56 to 22.68 m^2^ g^−1^, together with an enhancement in BJH pore volume from 0.000371 to 0.048436 cm^3^ g^−1^. This improvement in surface area and pore volume can be ascribed to flaky nanowalls of Ni(OH)_2_, which could benefit to the fast charge/discharge kinetic of Ni(OH)_2_ electrode^7^,^21^. Moreover, we note that the enhanced surface area and pore volume occur in the sample prepared from the smaller amount of ammonia added. For example, the sample of A-3/CFP shows a relatively higher surface area of 22.68 cm^2^ g^−1^ together with a higher BJH deposition cumulative pore volume of 0.048436 cm^−3^ g^−1^, as compared with those of A-4/CFP and A-6/CFP.

**Figure 3:**
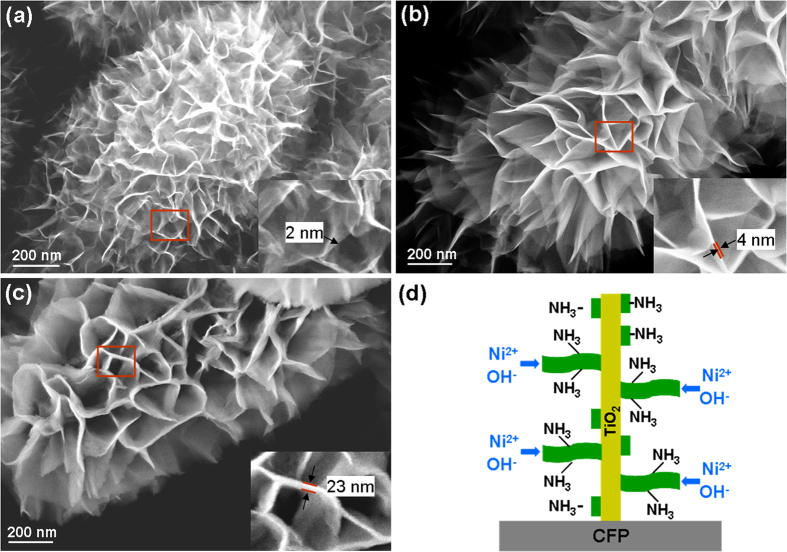
SEM images of (a) A-3/CFP, (b) A-4/CFP, (c) A-6/CFP and the insets show the high-magnified SEM images of Ni(OH)_2_. (**d**) Schematic diagram showing the Ni(OH)_2_ nanowalls growth process.

**Figure 4:**
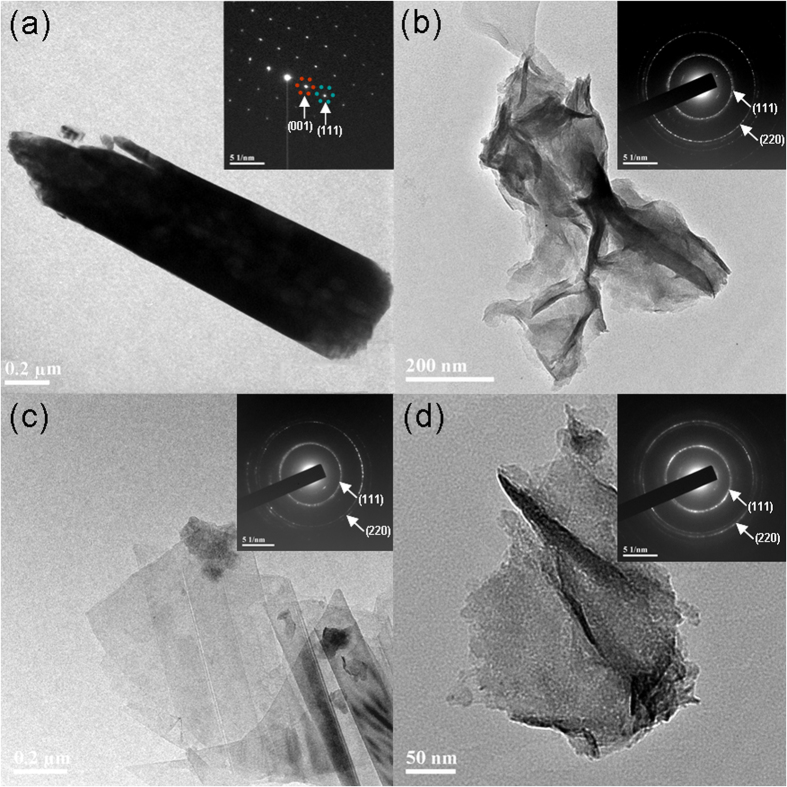
TEM images and SAED patterns of (a) TiO_2_ nanowire, (b) Ni(OH)_2_ flakes, A-3/CFP, (c) A-4/CFP and (d) A-6/CFP.

Figure 3 shows the SEM images of A-3/CFP, A-4/CFP and A-6/CFP, respectively, where the morphologies of Ni(OH)_2_ nanoflakes are different. For the sample prepared from a smaller amount of ammonia added (i.e., A-3/CFP), gauzy Ni(OH)_2_ sheets with curved surfaces are observed and the wall thickness of the nanosheet in A-3/CFP is estimated to be ~2 nm. With the increasing amount of ammonia added, the Ni(OH)_2_ nanosheets become flat and the wall thickness increases to ~23 nm in A-6/CFP. The core-shell structures and phases of TiO_2_@Ni(OH)_2_ were further elucidated by the transmission electron microcopy (TEM) and selected area electron diffraction (SAED) analysis. Figure 4a shows a typical TEM image of an individual TiO_2_ nanowire and the inset shows the SAED pattern recorded from this particle. The bright and sharp diffraction spots of rutile TiO_2_ (JCPDF 65-0192) are clarified, an indication of the single-crystalline nature of the TiO_2_ nanowrie with (001) orientation^29^. A detailed morphology and structure of nanoflakes from the shell material of Ni(OH)_2_ are shown in Fig. 4b–d. In consistence with the SEM studies, nanosheets in A-3/CFP exhibit a curved thin flaky appearance, while the sample prepared from the higher concentration of ammonia show thick and flat nanosheet layers of Ni(OH)_2_. The SAED patterns of the three samples are almost identified, as shown in the insets, where similar ring images are observed. These rings are indicative of a polycrystalline nature, and they correspond to the (111) and (220) planes of Ni(OH)_2_, respectively (JCPDS 47-1049).

## Discussion

In order to understand the growth mechanism, the chemical reaction process of Ni(OH)_2_ in the solution was systemically investigated. The general formation mechanism for sphere-shaped hydroxide particles in the presence of ammonia has been discussed previously. It has been suggested that the metal ions coordinate to the ammonia first to form a starting salt, which subsequently slowly releases to the basic solution to yield hydroxide particles^30^,^31^,^32^. The detailed process can be briefly described as follows:


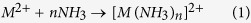



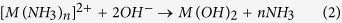


In our case of the nanostructured Ni(OH)_2_, the hydrolysis of nickel-ammonia complex of [Ni(NH_3_)_6_]^2+^ has been proposed^23^. Together, ammonia and NiSO_4_ form [Ni(NH_3_)_6_]^2+^ and then hydrolyze into Ni(OH)_2_ subsequently. These steps are controlled by the ammonia added into the solution and the detailed chemical reactions are given below:


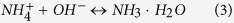



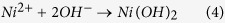



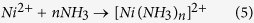



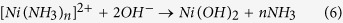


From the reaction processes above, there are dual roles played by ammonia. One is that the OH^−^ released from the ammonia will react with Ni^2+^ to form Ni(OH)_2_. On the other hand, NH_3_ in ammonia will consume part of Ni^2+^ to generate [Ni(NH_3_)_6_]^2+^, which will be hydrolyzed into Ni(OH)_2_ with the participation of extra OH^−^ 23.

Two different formation mechanisms of Ni(OH)_2_ sheets have been proposed: “oriented crystal growth” and “self-assembly”. In the latter, it was proposed that the adjacent Ni-based hydroxide particles tend to self-organize at a planar interface to show a common crystallographic orientation^33^. In the “oriented crystal growth” theory, it argues that the oriented growth of Ni(OH)_2_ originates from the NH_3_ molecule selectively adsorbs on certain crystal phases of Ni(OH)_2_, which therefore suppress the growth of Ni(OH)_2_ along this particular direction. However, we noted that both hypotheses share a common understanding that the formation of the hydroxides starts from heterogeneous nucleation process. In our case, the formation of Ni(OH)_2_ can be explained by heterogeneous nucleation and subsequent oriented crystal growth. The TiO_2_ nanowires acting as backbones provide sites for the preferential deposition of Ni-based hydroxides, and the growth process of the shell structure is proposed and shown schematically in Fig. 3d. At the beginning of the growth process, Ni^2+^ ions first react with OH^−^ released from the ammonia to form Ni(OH)_2_ nanocrystal seeds, which anchor to the surface of the TiO_2_ nanowire to reduce the surface energy. With the increase in reaction time, 2D Ni(OH)_2_ is formed due to the NH_3_ molecules that selectively adsorb on the surface of Ni(OH)_2_ nanosheets by hydrogen bonds, which exist between the nitrogen atom and the hydrogen atom of the surface hydroxyl groups (N-H-O)^23^. The NH_3_ then suppresses the growth of Ni(OH)_2_ along out-of-surface direction, but enables in-plane sheets growth. It is also mentioned that K_2_S_2_O_8_ plays a critical role in the formation of nanoflake shell, where it functions as an oxidant that drive the whole reaction and facilitate the heterogeneous nucleation in the chemical solution. Without the participation of K_2_S_2_O_8_, the nanoflake shell can not be generated on TiO_2_ scaffold^33^.

Based on the above understanding of the growth process for Ni(OH)_2_ nanosheets, the modification of wall thickness of Ni(OH)_2_ by ammonia can be well explained. NH_3_ molecules adsorbed on the surface of Ni(OH)_2_ nanosheets will react with Ni^2+^ in the solution to form [Ni(NH_3_)_6_]^2+^, which can be further translated into Ni(OH)_2_ upon reaction with OH^−^ as illustrated in Equ. (6). An increase in the concentration of OH^−^ accompanied with more ammonia added helps the hydrolysis of [Ni(NH_3_)_6_]^2+^ into Ni(OH)_2_ effectively, and therefore resulting in an increase in the nanowall thickness. In the present work, when the amount of ammonia is increased from 3 to 6ml, the wall thickness of Ni(OH)_2_ is increased from 2 to 23 nm.

**Figure 5:**
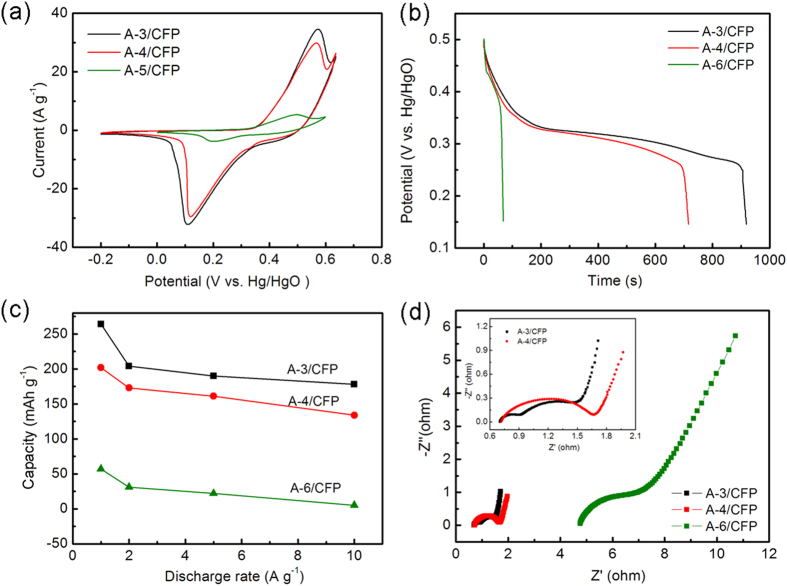
Comparisons of the electrochemical performance of the three samples: (**a**) CV curves ranging from −0.2 to 0.6 V at a scant rate of 5 mVs^−1^, (**b**) charging-discharging curves at 1 A g^−1^, (**c**) capacity at different current densities, (**d**) impedance Nyquist plots between 0.001 and 100 kHz at 5 mV applied voltage.

The electrochemical behavior of the three TiO_2_@Ni(OH)_2_ core-shell nanostructures are evaluated in a three-electrode measurement system by using the active material as the working electrode, the platinum foil as the counter electrode, and Hg/HgO electrode as the reference electrode. As shown in Fig. 5a, the cyclic voltammogram (CV) curves are recorded at a scan rate of 5 mV s^−1^ with a potential window ranging from -0.2 to 0.6 V. One notes that the area enclosed by the CV curve is decreased for the sample prepared from the larger amount of ammonia added. For instance, The CV curve of A-3/CFP (black line) generates an area larger than that of the A-6/CFP (green line), demonstrating an enhanced charge storage capability. Moreover, one set of sharp redox peaks is observed, which reveals that the capacity characteristics are governed mainly by the faradaic redox reaction, rather than electric double layer capacitors (EDLC) with CV curves in a rectangular shape^34^. The electrochemical charge transfers in Ni(OH)_2_ are along the chain of Ni (II)-Ni(III) in the basic electrolyte and the reaction can be described as follows^35^


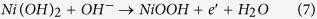


The charging-discharging curves of the three TiO_2_@Ni(OH)_2_ core-shell nanostructures are shown in Fig. 5b, at a current density of 1 A g^−1^. It indicates that the charging-discharging steps become more obvious for the sample prepared from the smaller amount of ammonia added (e.g. A-3/CFP) and the corresponding discharging time is dramatically extended. This demonstrates an improved electrochemical performance of battery type Ni(OH)_2_ electrode in a hybrid supercapacitor device through decreasing the concentration of ammonia.

The variation of capacity at different current densities is shown in Fig. 5c, where a nearly stable capacity value at high current densities is clarified. It indicates a desirable retention capability, resulting from the 3D nanostructure supported by the TiO_2_ scaffold, which provides plenty of conductive tunnels for an efficient charge transfer^36^. The electrochemical performance is improved for the TiO_2_@Ni(OH)_2_ prepared from the lower concentration of ammonia. As shown in Fig. 5c, the core-shell nanowire arrays of A-3/CFP exhibit a specific capacity of 264 mAh g^−1^ at 1 A g^−1^ and 178 mAh g^−1^ at 10 A g^−1^. In contrast, with the increasing amount of ammonia added, the sample shows a lower storage capability (e.g., the A-6/CFP delivers a much lower capacity of 57 mAh g^−1^ at 1 A g^−1^ and 5 mAh g^−1^ at 10 A g^−1^). Moreover, the electrochemical performance demonstrated for the core-shell arrays of TiO_2_@Ni(OH)_2_ in this work is much better than those of several other nanostructured NiO films^37^,^38^,^39^, TiO_2_-NiO counterpart^40^ and NiO powders^41^,^42^. The observed structure-property relationship can well be ascribed to the thinner nanowall and higher surface area, which provides more sites for the redox reaction, for the Ni(OH)_2_ derived from the lower ammonia concentration^43^,^44^.

EIS is measured and the corresponding Nyquist plots are shown in Fig. 5d. The electrochemical impedance measurement was biased to 0 V (vs. the open-circuit voltage) using the three-electrode system, at a frequency ranging from 0.01 to 100 kHz with 5 mV RMS voltage perturbation amplitude. The impedance spectra of the electrodes made of TiO_2_@Ni(OH)_2_ nanowire arrays exhibit similar semicircles in the high frequency region and straight line in the low frequency region. It is noted that the spike-like region is more close to the imaginary axis for the sample prepared from the smaller amount of ammonia added (A-3/CFP), demonstrating fast charge-transfer kinetics and electric responses resembling a circuit with low resistance and large capacitance connected in parallel^45^. In contrast, in the impedance curve of A-6/CFP, there is indication of the mass diffusion obstacles for ion or charge transfer in the electrode. In addition, it is also noted that the serial resistance (intercept on real axis) and charge transfer resistance (diameter of the semicircle) of A-3/CFP are lower than those of the A-4/CFP, followed by those of A-6/CFP. The serial resistance is ascribed to the inter-granular electronic resistance between active material particles, and the contact resistance between the active material and current collector, while the charge transfer resistance originates at the electrode/electrolyte interface^46^,^47^. The EIS data clearly demonstrates that the TiO_2_@Ni(OH)_2_ prepared from the smaller amount of ammonia added to the solution gives rise to improved conductivity in both serial and charge transfer parts. It therefore leads to enhanced electron transport efficiency and contributes greatly to the observed improvement in capacity of A-3/CFP.

**Figure 6:**
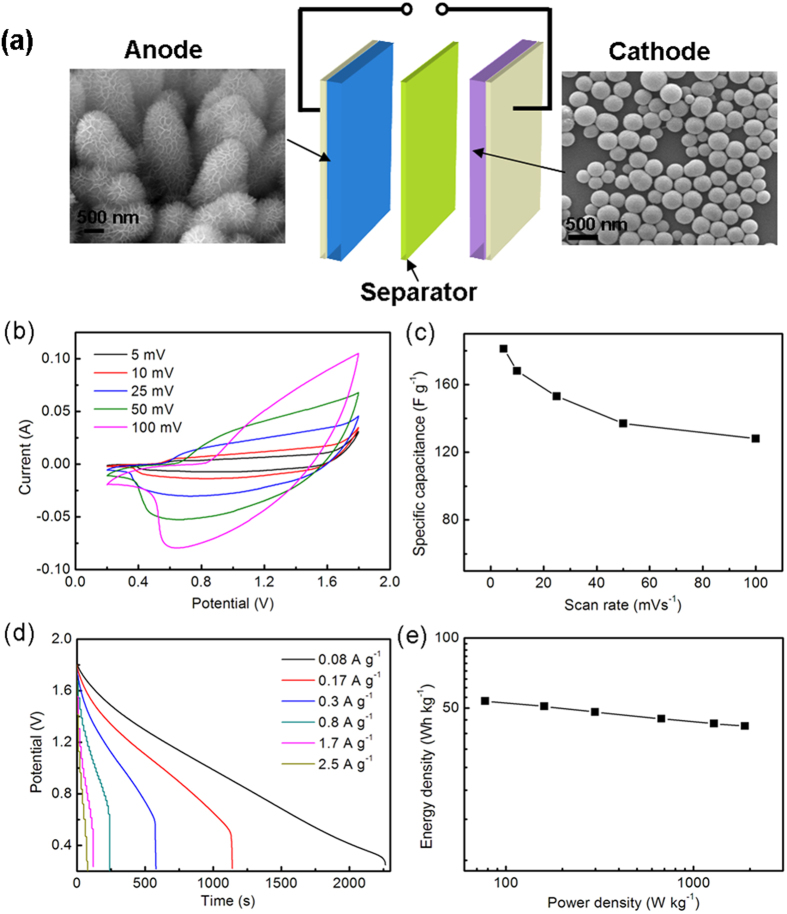
(**a**) Schematic illustration of the A-3//MCs asymmetric supercapacitor prototype with 6 M KOH as the electrolyte. (**b**) CV curves of A-3//MCs at different scan rates. (**c**) Specific capacitances at different scan rates. (**d**) Discharge curves of A-3//MCs asymmetric device at different current densities. (**e**) Ragone plot of the A-3//MCs asymmetric device.

To evaluate the capacitive performance for a full-cell device, the A-3/CFP electrode-based asymmetric supercapacitor (ASC) prototype is chosen^8^,^48^. As shown in Fig. 6a, TiO_2_@Ni(OH)_2_/CFP was chosen as the anode electrode and the mesoporous carbon (MCs)-based electrode was chosen as the cathode. For convenience, the asymmetric supercapacitor is labeled as A-3//MCs, in which the mass ratio of the anode to cathode active materials is around 1:5. CV curves were measured at various scan rates with voltage windows ranging from 0.2 to 1.8 V. It shows a rectangular-like shape in CV curves, indicating a similar capacitive behavior as those of EDLCs and RuO_2_. The specific capacitances calculated as a function of scan rates are summarized in Fig. 5c. The maximum specific capacitance is 181 F g^−1^ at 5 mV s^−1^, which is substantially higher than those recently reported for the Co_3_O_4_@Ni(OH)_2_//graphene (110 F g^−1^)^21^. Moreover, the ASC device demonstrates a good rate retention, with 70% of capacitance retained upon increasing the scan rate from 5 to 100 mV s^−1^.

To further evaluate the device performance, galvanostatic discharge measurements at various charge-discharge current densities were conducted. As shown in Fig. 6d, there is a nearly linear behavior in the discharge of A-3//MCs, which is an indication of capacitor-like behavior^21^. Both power density and energy density are important parameters for the performance of supercapacitor devices^49^. Ragone plot calculated from the charging-discharging curves is shown in Fig. 6e. At the low current density of 0.1 A g^−1^, the energy density and power density are 53.54 Wh kg^−1^ and 77.71 W kg^−1^, respectively, for A-3//MCs. At the high discharge current density of 2.5 A g^−1^, the energy density remains at 41.78 Wh kg^−1^ at the power density of 1881.87 Wkg^−1^. The superior energy density measured for the TiO_2_@Ni(OH)_2_/CFP-based asymmetric device is in part attributed to the high specific capacitance of the electrode material, which can be optimized by modulating nanostructure of Ni(OH)_2_ nanoflakes.

## Conclusions

3D TiO_2_@Ni(OH)_2_ core-shell nanostructures are successfully made by the combination of hydrothermal reaction and chemical bath deposition. By tuning the amount of ammonia added into the solution, the density and morphology of the Ni(OH)_2_ nanosheets can be well controlled. Flaky Ni(OH)_2_ with thin nanowall and high surface area is developed when a smaller amount of ammonia is added. The growth process of Ni(OH)_2_ is identified to involve heterogeneous nucleation and subsequent oriented crystal growth. There is a clear relationship between the nanostructural feature and electrochemical performance for the 3D TiO_2_@Ni(OH)_2_ core-shell arrays. Both the energy storage capability and conductance are improved in the sample with a larger surface area and smaller nanowall thickness. Superior capacity values of 264 mAh g^−1^ at 1 A g^−1^ and 178 mAh g^−1^ at 10 A g^−1^ are measured, when the interconnected shell structure of Ni(OH)_2_ is optimized, where there are fast ion/electron transfer and sufficient contact between the active material and electrolyte. An asymmetric prototype device is developed by employing TiO_2_@Ni(OH)_2_ as the anode, MCs as the cathode and 6 M KOH as the electrolyte. The asymmetric device shows superior capacitance behavior, including both high power density and energy density, demonstrating the potential for high performance supercapacitors.

## Methods

### Synthesis of self-supported TiO_2_/Ni(OH)_2_ core/shell nanowires

In our work, the self-supported TiO_2_ nanowire arrays on carbon fiber paper (CFP) were prepared by modifying a previously reported hydrothermal method^14^. In the typical process, the CFP was firstly cleaned with diluted HNO_3_ and deionized water for 5 min each, respectively. The Ti precursor was prepared by adding 1 ml of titanium butoxide to a well-mixed solution containing 15 mL of HCl and 15 mL of H_2_O. The mixture was then stirred for 24 h to obtain a clear solution. Afterward, a piece of clean CFP (2 × 2 cm^2^) was immersed into the above aqueous solution. Next, it was sealed into an autoclave and heated at 150 °C for 9 h, and then allowed to cool to room temperature spontaneously. After the reaction, the coated substrate was collected from the solution and then ultrasonicated in deionized water for 1 min to remove free nanoparticles and residual reactant. Finally, the nanowire coated CFP was calcinated at 550 °C in N_2_ for 2 h, completing the growth of TiO_2_ nanowire arrays on substrate. To grow Ni(OH)_2_ shells on the TiO_2_ nanowire cores, a solution was prepared by mixing 16 mL of 1M NiSO_4_·6H_2_O, 20 mL of 0.25 M K_2_S_2_O_4_ and different amounts of aqueous ammonia (24% NH_3_·H_2_O, 3 ml, 4 ml, 6 ml) in a Pyrex beaker at room temperature^19^. The TiO_2_-coated CFPs were immersed into the aqueous solution. When the room temperature chemical bath deposition of Ni(OH)_2_ was performed for 10 min, the Ni(OH)_2_-coated TiO_2_/CFP (TiO_2_@Ni(OH)_2_/CFP) sample was subjected to a high speed rotation at 500 rpm for another 5 min. The resultant samples were dried at 60 °C for 24 h.

### Material Characterization

The morphology and structure of each sample at every stages of preparation were characterized using scanning electron microscopy (SEM, Zeiss) and transmission electron microscopy (TEM, JEOL 2010) equipped with an energy-dispersive X-ray (EDS) analyzer. The specific surface area and pore volume were measured by N_2_ adsorption-desorption using the Brunauer-Emmett-Teller (BET, Micromeritics ASAP2020) method. X-ray diffraction (XRD) patterns of the samples were obtained with Bruker AXS (D8 Advance, Cu Kα, λ = 0.154060 nm) at 40 kV and 40 mA. The sample composition and structure were studied using a Kratos Axis Ultra X-ray photoelectron spectrograph (XPS, Kratos Analytical) equipped with a monochromatized Al Ka X-ray source, where the chamber pressure was controlled at 10^9^ Torr.

### Electrochemical Measurement

Electrochemical measurements of each electrode were conducted in three-electrode configuration with the as-prepared electrode as the working electrode, a platinum plate as the counter electrode, and an Hg/HgO as the reference electrode, in 6 M KOH electrolyte. The weight of each sample was measured using a Mettler Toledo X205DU microbalance (sensitivity, 0.01 mg; repeatability, 0.015 mg). Before weighing, all samples were dried in an oven at 120 °C for at least 1 day. The mass loading of the active material was calculated by m_2_-m_1_, where m_1_ is the mass of TiO_2_/CFP and m_2_ is the mass of the TiO_2_@Ni(OH)_2_/CFP. The loading of Ni(OH)_2_ is kept at around 5.2 mg cm^−2^. Electrochemical performance was evaluated by cyclic voltammetry (CV), galvanostatic charge-discharge and impedance spectroscopy by using Solartron System 1470E and 1400A, respectively. The electrochemical impedance measurement was biased to 0 V (vs. the open-circuit voltage) using the three-electrode system, at a frequency ranging form 0.001 to 100 kHz with a 5 mV RMS voltage perturbation amplitude. The device capacitance was calculated from the charging-discharging curve by the following equation: 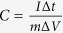, where *C* (F g^−1^) is the capacity, *I* (A) is the current, *ΔV* (V) is the potential window, *m* (g) is the mass of the active material and the *Δt* (s) is the discharging time.

## Additional Information

**How to cite this article**: Ke, Q. *et al.* 3D TiO_2_@Ni(OH)_2_ Core-shell Arrays with Tunable Nanostructure for Hybrid Supercapacitor Application. *Sci. Rep.* **5**, 13940; doi: 10.1038/srep13940 (2015).

## Figures and Tables

**Table 1:** BET surface area and BJH absorption cumulative pore volume of TiO_2_/CFP, A-3 /CFP, A-4 /CFP, and A-6 /CFP.

	BET surface area (m^2^g^−1^)	BJH absorption cumulative pore volume (cm^3^g^−1^)
TiO_2_/CFP	2.56	0.000371
A-3/CFP	22.68	0.048436
A-4/CFP	19.58	0.030831
A-6/CFP	15.87	0.028379

## References

[b1] Embden J. V. *et al.* Review of the synthetic chemistry involved in the production of core/Shell semiconductor nanocrystals. Aust. J. Chem. 60, 457–471 (2007).

[b2] Agarwal R. Heterointerfaces in semiconductor nanowires. Small 4, 1872–1893 (2008). 18932190 10.1002/smll.200800556

[b3] Law M., Goldberger J. & Yang P. D. Semiconductor nanowires and nanotubes. Annu. Rev. Mater. Res. 34, 83–122 (2004).

[b4] Pan H. & Feng Y. P. Semiconductor nanowires and nanotubes: effects of size and surface-to-volume ratio. ACS Nano. 2, 2410–2414 (2008). 19206409 10.1021/nn8004872

[b5] Cheng C. *et al.* Hierarchical sssembly of ZnO nanostructures on SnO_2_ backbone nanowires: low-temperature hydrothermal preparation and optical properties. ACS Nano. 3, 3069–3076 (2009). 10.1021/nn900848x 19772329

[b6] Cao L., Xu F., Liang Y. Y. & Li H. L. Preparation of the novel nanocomposite Co(OH)_2_/Ultra-stable Y zeolite and its application as a supercapacitor with high energy density. Adv. Mater. 16, 1853–1857 (2004).

[b7] Xia X. *et al.* Porous hydroxide nanosheets on preformed nanowires by electrodeposition: branched nanoarrays for electrochemical energy storage. Chem. Mater. 24, 3794–3799 (2012).

[b8] Tang Z., Tang C. & Gong H. A high energy density asymmetric supercapacitor from nano-architectured Ni(OH)_2_/Carbon nanotube electrodes, Adv. Funct. Mater. 22, 1272–1278 (2012).

[b9] Yan J., Khoo E., Sumboja A. & Lee P. S. Facile coating of manganese oxide on tin oxide nanowires with high-performance capacitive behavior, ACS nano, 4, 4247–4255 (2010). 20593844 10.1021/nn100592d

[b10] Liu J., Cheng C., Zhou W., Lia H. & Fan H. J. Ultrathin nickel hydroxidenitrate nanoflakes branched on nanowire arrays for high-rate pseudocapacitive energy storage. Chem. Commun. 47, 3436–3438 (2011). 10.1039/c0cc04906a 21286657

[b11] Lu X. *et al.* H-TiO_2_@MnO_2_//H-TiO_2_@C core–shell nanowires for high performance and flexible asymmetric supercapacitors. Adv. Mater. 25, 267–272 (2013). 23080535 10.1002/adma.201203410

[b12] Liao J.-Y. *et al.* Multifunctional TiO_2_-C/MnO_2_ Core–double-shell nanowire arrays as high-performance 3D electrodes for lithium ion batteries. Nano Lett. 13, 5467–5473 (2013). 24079359 10.1021/nl4030159

[b13] Xia X. *et al.* Integrated photoelectrochemical energy storage: solar hydrogen generation and supercapacitor. Sci. Rep. 2, 981 (2012). 23248745 10.1038/srep00981 PMC3522068

[b14] Wang G. M. *et al.* Hydrogen-treated TiO_2_ nanowire arrays for photoelectrochemical water splitting. Nano Lett. 11, 3026–3033 (2011). 21710974 10.1021/nl201766h

[b15] Zhou Q. *et al.* High Rate Capabilities of NiCo_2_O_4_-Based Hierarchical Superstructures for Rechargeable Charge Storage J. Electrochem. Soc. 161, 1922–1926 (2014).

[b16] Brousse T., Bélanger D. & Long J. W. To be or not to be pseudocapacitive. J. Electrochem. Soc. 162, 5185–5189 (2015).

[b17] Wu H. *et al.* Aligned NiO nanoflake arrays grown on copper as high capacity lithium-ion battery anodes. J. Mater. Chem. 22, 19821–19825 (2012).

[b18] Wang G. *et al.* Free-standing nickel oxide nanoflake arrays: synthesis and application for highly sensitive non-enzymatic glucose sensors. Nanoscale 4, 3123–3127 (2012). 22491751 10.1039/c2nr30302g

[b19] Li G. Wang X., Ding H. & Zhang T. A facile synthesis method for Ni(OH)_2_ ultrathin nanosheets and their conversion to porous NiO nanosheets used for formaldehyde sensing. RSC Advances 2, 13018–13023 (2012).

[b20] Li J., Zhao W., Huang F., Manivannan A. & Wu N. Single-crystalline Ni(OH)_2_ and NiO nanoplatelet arrays as supercapacitor electrodes. Nanoscale 3, 5103–5109 (2011).

[b21] Tang C., Yin X. & Gong H. Superior performance asymmetric supercapacitors based on a directly grown commercial mass 3D Co_3_O_4_@Ni(OH)_2_ core–shell electrode. ACS Appl. Mater. Interfaces 5, 10574–10582 (2013). 24090480 10.1021/am402436q

[b22] Dubal D. P., Fulari V. J., & Lokhande C. D. Effect of morphology on supercapacitive properties of chemically grown β-Ni(OH)_2_ thin films. Microporous Mesoporous Mater. 151, 511–516 (2012).

[b23] Ni X. *et al.* High-yield synthesis of nickel flowers from nickel hydroxide precursor. Chem. Lett. 34, 1408–1409 (2005).

[b24] Wang Y., Zhu Q. & Zhang H. Fabrication of β-Ni(OH)_2_ and NiO hollow spheres by a facile template-free process. Chem. Commun. 41, 5231–5233 (2005). 10.1039/b508807k 16228045

[b25] Li. G. *et al.* Controllable synthesis of 3D Ni(OH)_2_ and NiO nanowalls on various substrates for high-performance nanosensors. Small 10.1002/smll.201400830 25273523

[b26] Subramanian P. *et al.* Preparation of reduced graphene oxide-Ni(OH)_2_ composites by electrophoretic deposition: application for non-enzymatic glucose sensing. J. Mater. Chem. A 2, 5525–5533 (2014).

[b27] Yan H. *et al.* Graphene homogeneously anchored with Ni(OH)_2_ nanoparticles as advanced supercapacitor electrodes. CrystEngComm, 15, 10007–10015 (2013).

[b28] Tang C., Yin X. & Gong H. A study on dramatically enhanced capacitance of graphene-decorated hierarchically porous nickelian heterogenite for energy storage application. Electrochim. Acta 114, 543–550 (2013)

[b29] Wu J. B., Guo R. Q., Huang X. H. & Lin Y. Construction of self-supported porous TiO2/NiO core/shell nanorod arrays for electrochemical capacitor application. J. Power Sources 243, 317–322 (2013).

[b30] Junichi I., Yuri K., Tetsushi M. Toyoshi, investors; Tanaka Chemical Corporation, Original assignee. Nickel hydroxide particles having an α- or β-cobalt hydroxide coating layer for use in alkali batteries and a process for producing the nickel hydroxide. United States patent US 6,040,007. 2000 Mar 21.

[b31] Lee M. H., Kang Y. J., Myung S. T. & Sun Y. K. Synthetic optimization of Li[Ni_1/3_Co_1/3_Mn_1/3_]O_2_ via co-precipitation. Electrochim. Acta 50, 939–948 (2004).

[b32] Cho J. LiNi_0.74_Co_0.26-x_Mg_x_O_2_ cathode material for a Li-Ion cell. Chem. Mater. 12, 3089–3094 (2000).

[b33] Xia X. *et al.* High-quality metal oxide core/Shell nanowire arrays on conductive substrates for electrochemical energy storage. ACS nano, 6, 5531–5538 (2012). 22545560 10.1021/nn301454q

[b34] Zhang L. *et al.* Highly conductive and porous activated reduced graphene oxide films for high-power supercapacitors. Nano Lett. 12, 1806–1812 (2012). 22372529 10.1021/nl203903z

[b35] Xia X. H. *et al.* Hierarchically porous NiO film grown by chemical bath deposition via a colloidal crystal template as an electrochemical pseudocapacitor material. J. Mater. Chem. 21 671–679 (2011).

[b36] Cai Y. *et al.* Transition metal atoms pathways on rutile TiO_2_ (110) surface: Distribution of Ti^3+^ states and evidence of enhanced peripheral charge accumulation. J. Chem. Phys. 138, 154711 (2013). 23614440 10.1063/1.4801025

[b37] Wu M. S., Huang Y. A., Yang C. H. & Jow H. H. Electrodeposition of nanoporous nickel oxide film for electrochemical capacitors. Int. J. Hydrogen Energy 32, 4153–4159 (2007).

[b38] Zhang Y. Q. *et al.* Self-assembled synthesis of hierarchically porous NiO film and its application for electrochemical capacitors. J. Power Sources 199, 413–417 (2012).

[b39] Xia X. H. *et al.* Graphene sheet/porous NiO hybrid film for supercapacitor applications. Chem. Eur. J. 17, 10898–10905 (2011). 21837714 10.1002/chem.201100727

[b40] Kim J. H. *et al.* Microstructure and pseudocapacitive properties of electrodes constructed of oriented NiO-TiO_2_ nanotube arrays. Nano Lett. 10, 4099–4104 (2010). 20873847 10.1021/nl102203s

[b41] Su D. W., Kim H. S., Kim W. S. & Wang G. X. Mesoporous nickel oxide nanowires: hydrothermal synthesis, characterisation and applications for lithium-ion batteries and supercapacitors with superior performance. Chem. Eur. J. 18, 8224–8229 (2012). 22589171 10.1002/chem.201200086

[b42] Wu M. S. & Hsieh H. H. Nickel oxide/hydroxide nanoplatelets synthesized by chemical precipitation for electrochemical capacitors. Electrochim. Acta 53, 3427–3435 (2008).

[b43] Zhu Y. *et al.* Ultrathin nickel hydroxide and oxide nanoshee: synthesis, characterizations and excellent supercapacitor performances, Sci. Rep. 4, 5787 (2014). 25168127 10.1038/srep05787 PMC4148663

[b44] Zhu Y. *et al.* Surface-enabled superior lithium storage of highquality ultrathin NiO nanosheets, J. Mater. Chem. A, 2, 7904 (2014).

[b45] Lai L. *et al.* Tuning graphene surface chemistry to prepare graphene/polypyrrole supercapacitors with improved performance. Nano Energy, 1, 723–731 (2012).

[b46] Portet C., Taberna P. L., Simon P. & Laberty-Robert C. Modification of Al current collector surface by sol-gel deposit for carbon-carbon supercapacitor applications. Electrochim. Acta. 49, 905–912 (2004).

[b47] Girija T. C. & Sangaranarayanan M. V. Analysis of polyaniline-based nickel electrodes for electrochemical supercapacitors. J. Power Sources. 156, 705–711 (2006).

[b48] Guan C. *et al.* Iron Oxide-Decorated Carbon for Supercapacitor Anodes with Ultrahigh Energy Density and Outstanding Cycling Stability, ACS Nano. 9, 5198–5207 (2015). 25868870 10.1021/acsnano.5b00582

[b49] Zhang L. & Gong H. Improvement in flexibility and volumetric performance for supercapacitor application and the effect of Ni0-Fe ratio on electrode behaviour, J. Mater. Chem. A. 3, 7607–7615 (2015).

